# The HPV-DNA Test in Pregnancy: A Review of the Literature

**DOI:** 10.7759/cureus.38619

**Published:** 2023-05-06

**Authors:** Nektaria Zagorianakou, Ioannis Mitrogiannis, Kyriakos Konis, Stylianos Makrydimas, Leonidas Mitrogiannis, George Makrydimas

**Affiliations:** 1 Cytology, University of Ioannina, Ioannina, GRC; 2 Obstetrics & Gynecology, General Hospital of Arta, Arta, GRC; 3 Medical School, Aristotle University of Thessaloniki, Thessaloniki, GRC; 4 Anatomy, Histology, & Embryology, University of Ioannina, Ioannina, GRC; 5 Obstetrics & Gynecology, University Hospital of Ioannina, Ioannina, GRC

**Keywords:** hpv diagnosis, hpv infection, cervical cancer screening, pregnant women, pregnancy, hpv-dna test

## Abstract

Human papillomavirus (HPV) is a virtually necessary cause of cervical cancer, and HPV genotypes are categorized either as high-risk or low-risk based on their potential to cause malignancy of the cervix. HPV-DNA detection is used widely for screening women at risk. However, its clinical significance is not proven sufficiently in pregnancy. The aim of this review was to summarize published data referring to the integration of the HPV-DNA test in cervical cancer screening during pregnancy. PubMed and Scopus were searched for articles investigating the HPV-DNA test during pregnancy as a primary association; greater interest was placed on studies published after 2000. Retrieved articles reported similarities or discrepancies in the HPV-DNA test in pregnant women compared to those who are not pregnant, its accuracy, and its integration in cervical cancer screening. The HPV-DNA test may constitute a helpful tool utilized for monitoring, risk stratification, and triage of cases that require colposcopy. If combined with the HPV-mRNA test, this might improve its specificity. However, when compared to HPV-DNA detection rates in non-pregnant women, the results were ambiguous, without giving the opportunity to draw safe conclusions. Both those findings and the high cost hold it back from wide use. Hence, the Papanicolaou smear (Pap smear) is still the first-line diagnostic tool and colposcopy-guided cervical biopsy is the “gold standard” method for the management of cervical intraepithelial neoplasia (CIN) treatment during pregnancy.

## Introduction and background

Human papillomavirus (HPV), a double-stranded DNA virus, is known to incorporate into epithelial cells and, under specific circumstances, trigger proliferation [1]. HPV infects various human tissue epithelial cells such as oral, genital, or skin mucosa, and its persistence could potentially lead to benign, precancer lesions and progression to cancer as well [2]. Over the years, at least 120 HPV genotypes have been identified, and almost half of them have been correlated to lower genital tract infections [1-3]. The association between HPV genotype and the prevalence of cervical cancer is used to specify them into two distinct subgroups: a) high-risk HPV (HR-HPV) oncogenic genotypes and b) low-risk HPV (LR-HPV) oncogenic genotypes. In regards to the HR-HPV subgroup, it is worth mentioning the following ones: 16, 18, 31, 33, 35, 39, 45, 51, 52, 56, 58, 59, 67, 68, and 70 [1,2].

An HPV infection underlies virtually all causes of cervical cancer, both in pregnant and non-pregnant women [1,4,5]; thus, vaccination strategies have been developed as a prevention tool. The prevalence of cervical cancer in pregnant women ranges between 0.80 and 1.50 cases out of 10,000 births and one to three of the 100 women diagnosed with cervical cancer are pregnant or in the postpartum period at that time. Cervical cancer is high in the ranks of malignancies diagnosed during pregnancy [6,7]. Pregnancy is a period of time in which hormonal and immunological alterations take place, raising the question of whether pregnancy affects the natural history of HPV. Although contradictory statements were formulated in the past in relation to HPV infection status during pregnancy, it is now clearly clarified that pregnancy does not affect the medical prognosis of the disease [1,5,8-10]. HPV infections are more likely to suppress within nine to 15 months, but in some cases, may remain in the latent phase or even persist and progress [1]. The fact that some HPV oncogenic types are highly correlated to cervical intraepithelial neoplasia (CIN) and are a prerequisite step in cervical cancer pathogenesis urged the development and strongly supports the value of the HPV-DNA test in CIN screening procedures. Moreover, the HPV-DNA test is a valuable tool not only in the diagnosis but also in the follow-up period of patients with abnormal results or under treatment for CIN [3,11].

Our aim was to summarize data from published literature regarding the integration of the HPV-DNA test in cervical cancer screening and triage of pregnant women. To be more specific, our review presents relevant data to HPV-DNA testing during pregnancy compared to the non-pregnant population; regarding the sensitivity, specificity, contribution to screening strategies, and follow-up, as well. This is also an effort to highlight similarities or discrepancies in the HPV-DNA Test between pregnant and non-pregnant women. The aim of this review is to provide accurate up-to-date information to obstetricians and gynecologists wishing to acquire a total, representative, and clear view in terms of the HPV-DNA test during pregnancy.

## Review

Methods

A review of the literature for studies in the English language including data on the HPV-DNA test during pregnancy was conducted. The PubMed and SCOPUS databases were searched utilizing various combinations of the following terms: “HPV-DNA Test”, “Pregnant Women” AND “Pregnancy”. Our scientific interest focused especially on articles published after 2000, reflecting the current status of evidence. Titles and abstracts of the retrieved articles were scanned at first, but only the studies that were primarily designed to examine the association of the HPV DNA test in pregnant women were identified as the most relevant studies and were finally retrieved for full-text evaluation. The reference list of full-text reviewed articles was also scanned for relevant studies. During this process, all the articles referring to the HPV-DNA test during pregnancy were screened. All article types were eligible for inclusion such as clinical trials, case series, and case-control studies that examined the diagnostic value of the HPV-DNA test during pregnancy, described or assessed its integration in the cervical malignancy screening clinical routine, and compared those to the non-pregnant population. Studies referring solely to the HPV-DNA test in non-pregnant women were excluded. In addition, studies that used the HPV-DNA molecular test as a diagnostic tool aiming to examine another association, but not as a primary subject of investigation, were excluded too. Our search initially identified 438 results. The most relevant and representative studies that met our inclusion criteria were retrieved. Thus, 10 studies were included in the review reporting on the integration of the HPV-DNA test during pregnancy, a general comparison between pregnant and non-pregnant women, including similarities or discrepancies, and the potentially key role in triage for cervical cancer, either alone or in combination with HPV-mRNA tests. The characteristics of the included studies are presented in Table 1.

**Table 1:** Characteristics of included studies HPV: Human Papillomavirus; HR-HPV: High-Risk Human Papillomavirus; CIN: Cervical Intraepithelial Neoplasia; HSIL: High-Grade Squamous Intraepithelial Lesion; LSIL: Low-Grade Squamous Intraepithelial Lesion; ASC: Atypical Squamous Cells

First Author [Reference]	Title	Year of Publication	Study Design	Purpose	Population
Henrique Diorio de Souza [1]	Prevalence of oncogenic human papillomavirus in pregnant adolescents, association with colpocytological changes, risk factors, and obstetric outcomes	2022	Cross-sectional study	Impact of cervical oncogenic HPV detection in pregnant adolescents, its prevalence and risk factors	303 pregnant adolescents
Yue He [3]	High-risk human papillomavirus management in pregnancy with cervical intraepithelial neoplasia during pregnancy and postpartum in China	2014	Prospective case-control study	Relationship between CIN and HR-HPV during pregnancy and postpartum	168 pregnant women
Paul K.S. Chan [10]	Prevalence and genotype distribution of cervical human papillomavirus infection: comparison between pregnant women and non-pregnant controls	2002	Cross-sectional study	Compare the prevalence and genotype distribution of cervical HPV infection between pregnant and non-pregnant women	308 pregnant women and 308 non-pregnant women
Danielle W. Lu [12]	Prevalence and typing of HPV DNA in atypical squamous cells in pregnant women	2003	Clinical Trial	Prevalence and typing of HPV-DNA in pregnant women with ASC diagnosis and assessment of pregnancy-related changes contribution to ASC diagnosis	Pregnant women
Achim Schneider [13]	Increased prevalence of human papillomaviruses in the lower genital tract of pregnant women	1987	Clinical Trial	Evaluate the statement that pregnancy triggers HPV replication via the determination of HPV infection and replication rates in pregnant women versus non-pregnant	92 pregnant women vs 96 non-pregnant women
Y. Aydin [14]	Prevalence of human papilloma virus infection in pregnant Turkish women compared with non-pregnant women	2010	Clinical Trial	HPV prevalence aiming to define 100 genotypes and the subset of 14 oncogenic genotypes in pregnant Turkish women and compare them with non-pregnant women	164 pregnant women and 153 non-pregnant women
Carlos Hernandez-Giron [15]	High-risk Human papillomavirus detection and related risk factors among pregnant and nonpregnant women in Mexico	2005	Cross-sectional study	Identify differences in the prevalence of HPV infection between pregnant and nonpregnant women; and investigate the association of a positive HPV-DNA with various factors	278 pregnant women and 1060 non-pregnant women
Khanuja Esha [16]	A study of cervical intraepithelial neoplasia in pregnancy	2014	Prospective pilot study	Incidence of HPV infection and CIN in pregnant women and compare the Pap smear with the HPV-DNA test in detecting HPV infection	Pregnant women
Antonio Frega [17]	Expression of E6/E7 HPV-DNA, HPV-mRNA, and colposcopic features in management of CIN2/3 during pregnancy	2016	Clinical Trial	Assess the management of abnormal cytology (LSIL, HSIL) during/ after pregnancy and the predictive role of HPV molecular tests	500 pregnant women
Yong-Mei Yang [18]	Preliminary study of the use of E6/E7 mRNA detection in screening and triage management of HR-HPV infection during pregnancy	2021	Preliminary study	Assess the application of E6 and E7 mRNA detection in the diagnosis and management of HR-HPV infection for high-grade cervical lesions during pregnancy	1058 pregnant women

Pregnant versus non-pregnant women

Several studies assessed HPV-DNA test results in both pregnant and non-pregnant women in the past. Chan et al. found that there is no statistically significant difference in HPV-DNA detection rates between those two groups [10]. To be more specific, this study showed that 10.1% of pregnant women and 11.4% of non-pregnant women had an HPV-DNA positive test. It was also highlighted that the majority of pregnant women detected with a positive HPV-DNA had a normal Papanicolaou smear (Pap smear) result previously and gestational age did not alter the HPV-DNA detection rates. In addition, Lu et al. study detected similar rates of positive HPV-DNA between pregnant and non-pregnant women with atypical squamous cells (ASC) (88.6% and 83.8%, respectively) [12].

In contrast, a study conducted by Schneider et al. suggested that pregnancy is either a risk factor for HPV or increases its detection rates due to increased proliferation [13]. Twenty-eight percent (28%) of pregnant women had a positive HPV-DNA test with a normal Pap smear compared to almost 12% of non-pregnant women with a positive HPV-DNA test. This study also reported that an HPV-DNA positive test is more likely to be detected in the second half of pregnancy. Schneider et al. found that the HPV 16 genotype was more common in the group consisting of pregnant women [13] while Chan et al. showed that HPV 16 and 58 were the most common genotypes in both pregnant and non-pregnant women [10].

Souza et al. conducted a study in Brazil including 303 adolescent women during pregnancy and found that 50.5% were detected with HR-HPV [1], but several other studies suggest that this rate varies between 5.8% and 51.7% [9,10,19]. This study also showed that low-grade squamous intraepithelial lesions (LSIL) and atypical squamous cells of undetermined significance (ASCUS) were associated with HR-HPV. Nevertheless, Souza et al. suggest that both HPV infection and cervical lesions tend to regress [1]. In addition, Aydin et al. in their study support that HR-HPV prevalence in pregnant women (14.6%) compared to non-pregnant women (9.8%) living in Turkey is statistically significantly different. HPV genotypes 16 and 18 were detected at a more frequent rate during pregnancy with a statistically significant difference [14].

In agreement with previous studies, Hernadez-Giron et al. in their study claim that HPV-DNA detection rates during pregnancy are higher than those rates in non-pregnant women [15]. It is also reported that the detection rates of HPV-DNA do not support a clear correlation to gestational age, which is controversial with the findings of Schneider et al. [13]. Moreover, Hernadez-Giron et al. also claim that 13 HR-HPV genotypes were most commonly detected in pregnancy-related groups [15].

HPV-DNA test integration during pregnancy

The HPV-DNA test is integrated into the cervical cancer screening procedure alone or in combination with a Pap smear [16,20], but there has been a lot of research in order to reach this conclusion. The HPV-DNA test has higher sensitivity, lower specificity, and higher negative predictive value (NPV) in comparison to the Pap smear [16], but it is reasonable to wonder whether pregnancy might affect HPV-DNA test components.

A study conducted by Khanuja et al. tested pregnant women for HPV infection via a Pap smear and the HPV-DNA test as well [16]. It was found that 18% of pregnant women had a positive HPV-DNA test. None of the pregnant women with a negative HPV-DNA test showed results in the Pap smear such as koilocytic changes but six pregnant women with koilocytic changes shown in the Pap smear had also an HPV-DNA positive test result. In addition, Khanuja et al. reported that the majority of Pap smear results had the presence of inflammatory signs, both in HPV-DNA positive and negative pregnant women [16]. Nevertheless, two cases of the HPV-DNA positive group had a normal Pap smear result and one case had an inadequate sample. They conclude that the HPV-DNA test is the most sensitive and reliable technique in detecting HPV infection while a Pap smear will identify HPV infection at the moment that women reach its clinically infective stage. Taking into consideration the high cost of the HPV-DNA technique when compared to a Pap smear, they suggest that antenatal women should be tested with a Pap smear in clinical routine [16].

He et al. support that 82.1% of pregnant women cases with either various CIN grades or cervicitis had an HPV-DNA positive test [3]. The higher the CIN grade, the higher the positive HPV-DNA test rates in those groups, respectively. They also found that 39.1% of pregnant women with positive HPV-DNA tests regressed and turned out to have a negative HPV-DNA test during pregnancy or three to six months postpartum. Moreover, another observation was that the higher the CIN grade, the lower the HPV-DNA test rates that naturally changed to negative during pregnancy or three to six months postpartum. Those statements support the fact that it is harder for the HPV infection to be removed in cases with higher CIN grades. Although the quantity of HPV-DNA could not be associated with CIN grade with a statistically significant difference, it is found that CIN III has higher HPV-DNA quantity compared to other CIN grades, three to six months postpartum. He et al. finally suggest that HPV-DNA test should be routinely conducted during pregnancy and three to six months postpartum for pregnant women with CIN; and rates of HPV-DNA test that changes to negative in the postpartum should be carefully considered for CIN III cases as well [3]. Nevertheless, colposcopy-guided cervical biopsy is still the “gold standard” method to diagnose CIN grade three to six months postpartum, and define treatment management [3].

Another pilot study conducted by Lu et al. showed a high incidence of HPV-DNA positive tests during pregnancy while the Pap smear of those cases did not report an abnormal result [12]. This observation is confusing regarding the clinical management of those cases, but it is suggested that an HPV-DNA positive test result in pregnant women should not alter the negative Pap smear diagnosis. However, it might be a sign indicating closer follow-up to pregnant women with an HPV-DNA positive test and a history of normal Pap smear results [12]. On the other hand, a combination of negative HPV-DNA test and normal Pap smear results in those women reduces dramatically the risk of cervical cancer, and they should follow cervical cancer screening guidelines for the general population. Lu et al., considering the findings in their study in combination with high HPV-DNA incidence of women at reproductive age, raised the ambiguity regarding the utility of HPV-DNA test contribution to the management of those cases [12].

The Frega et al. study reported on the accuracy of the HPV-DNA test during pregnancy and eight weeks postpartum in the detection of CIN II or CIN III cases [17]. Therefore, their findings regarding the HPV DNA test accuracy during pregnancy suggest the following: test sensitivity is 90.5%, test specificity is 67.9%, test NPV is 96.4% and test positive predictive value (PPV) is 43.2%. This study also evaluated HPV-mRNA accuracy regarding the detection of CIN II or CIN III in pregnant women and eight weeks postpartum. They found that the test sensitivity is 76.2%, test specificity is 98.7%, test NPV is 93.9%, and test PPV is 94.1%. A plausible explanation for the discrepancies regarding the sensitivity and specificity between those molecular tests is the fact that the mRNA targets a small number of HPV genotypes. Hence, HPV-mRNA may be utilized for risk stratification is those cases. Another study conducted by Yang et al. reported the results regarding the assessment of HPV-DNA and HPV-mRNA test accuracy in pregnant women with equal or greater to CIN II grade lesions [18]. To be more specific, HPV-DNA test sensitivity was 86.2%, specificity was 21.8%, PPV was 37.1% and NPV was 75%. On the other hand, HPV-mRNA test sensitivity was 65.5%, its specificity was 54.5%, PPV was 43%, and NPV was 75%. When those molecular tests were compared, it was found that the HPV-mRNA test has higher specificity but lower sensitivity than the HPV-DNA test. NPV and PPV did not present a statistically significant difference. They suggest that utilizing the HPV-mRNA test as a complementary tool to HPV-DNA positive test could improve the specificity in the diagnosis of equal or greater CIN II grade lesions, but this will not affect its sensitivity. This observation is based on the fact that none of these molecular tests is an effective screening tool alone. A unique report is made regarding HPV-DNA test specificity, which is not satisfactory and can lead not only to misunderstandings but also stress in pregnant women [18].

Discussion

Pregnancy is a period in a woman’s life when a variety of changes take place, which may potentially affect various human functions. In this case, a lot of things that are widely used in the general population, such as diagnostic tools, screening procedures, values of laboratory findings, and therapies, should be re-assessed and redefined in order to get integrated during pregnancy. The HPV-DNA test is widely used for cervical cancer screening in the general population [20], but its integration during pregnancy may differ in the general population and this should be evaluated. Results and relevant data of the included studies are presented in Table 2.

**Table 2:** Relevant data and results of the included studies HPV: Human Papillomavirus; HR-HPV: High-Risk Human Papillomavirus; CIN: Cervical Intraepithelial Neoplasia; LSIL: Low-Grade Squamous Intraepithelial Lesion; ASC: Atypical Squamous Cells; ASCUS: Atypical Squamous Cells of Undetermined Significance; PPV: Positive Predictive Value; NPV: Negative Predictive Value; Pap Smear: Papanicolaou Smear; N: Number

First Author [Reference]	Methods and Tools	Results and Notes
Henrique Diorio de Souza [1]	Pap smear, HPV DNA test	50.5% of the study population detected with HR-HPV; LSIL and ASCUS were correlated to HR-HPV (LSIL: 9.57% and ASCUS: 7.59% among adolescents); HPV infection and cervical lesions tend to regress rather than progress
Yue He [3]	Pap smear, HPV DNA test, Colposcopy	82.1% of pregnant women with CIN or cervicitis showed a positive HPV DNA test; 39.1% of pregnant women with a positive HPV DNA test regressed; The higher the CIN grade, the higher the positive HPV DNA test rates (CIN III: 97.6%, CIN II: 86.5%, CIN I: 76.8%); the higher the CIN grade, the lower the HPV DNA turning negative during pregnancy or postpartum (CIN III: 4.9%, CIN II: 46.9%, CIN I: 52%)
Paul K.S. Chan [10]	Pap smear, HPV DNA test	10.1% of pregnant women had a positive HPV DNA test versus 11.4% of non-pregnant women with a positive HPV DNA test; The majority of pregnant women with positive HPV DNA test showed a normal Pap smear; Gestational age didn’t affect HPV DNA detection rates; HPV 16 & 58 genotypes usually identified both in pregnant (N=15) and non-pregnant women (N=16)
Danielle W. Lu [12]	Pap smear, HPV DNA test	88.6% of pregnant women with ASC had a positive HPV DNA test versus 83.8% of non-pregnant women with ASC who had a positive HPV DNA test; 55% of pregnant women had a positive HPV DNA test, without evidence of abnormal Pap smear results.
Achim Schneider [13]	Pap smear, HPV-DNA test	28% of pregnant women had a positive HPV DNA test and normal Pap smear, while 12% of non-pregnant women had a positive HPV DNA test; Pregnancy is either a risk factor or increases detection rates for HPV; HPV DNA is usually detected in the second half of pregnancy; HPV 16 genotype is more common in pregnant women (occurred 3.9 times more frequently)
Y. Aydin [14]	Pap smear, HPV DNA test	14.6% of pregnant women had HR-HPV versus 9.8% of non-pregnant women with HR-HPV; HPV 16 & 18 genotypes are more frequent in pregnant women (12.1% in pregnant women versus 7.7% in non-pregnant women)
Carlos Hernandez-Giron [15]	HPV DNA test	Higher HPV DNA detection rates in pregnant women versus non-pregnant women; HPV DNA detection rates are not clearly correlated to gestational age (1^st^ Trimester: 41.2%, 2^nd^ Trimester: 28.3%, 3^rd^ Trimester: 42.6%); 13 HR-HPV genotypes were more common in pregnant women (37.1% in pregnant women versus 14.1% in non-pregnant women)
Khanuja Esha [16]	Pap smear, HPV DNA test	18% of pregnant women had a positive HPV DNA test; Six pregnant women with abnormal Pap smear results (such as koilocytic changes) showed a positive HPV DNA test; Pregnant women with a negative HPV DNA test had a normal Pap smear (no koilocytic changes); HPV DNA is more sensitive and reliable, but a Pap smear should be used in everyday clinical routine
Antonio Frega [17]	Pap smear, HPV DNA test, HPV-mRNA test, Colposcopy	HPV DNA test in the detection of CIN II or CIN III cases during pregnancy and 8 weeks postpartum (Sensitivity= 90.5%, Specificity=67.9%, NPV=96.4%, PPV=43.2%); HPV-mRNA test in the detection of CIN II or CIN III cases during pregnancy and 8 weeks postpartum (Sensitivity=76.2%, Specificity=98.7%, NPV=93.9%, PPV=94.1%); HPV-mRNA should be used for risk stratification
Yong-Mei Yang [18]	Pap Smear, HPV DNA test, HPV-mRNA test, Colposcopy	HPV DNA test in the detection of equal or greater to CIN II grade lesions (Sensitivity=86.2%, Specificity=21.8%, PPV=37.1%, NPV=75%); HPV-mRNA test in the detection of equal or greater to CIN II grade lesions (Sensitivity=65.5%, Specificity=54.5%, PPV=43%, NPV=75%); HPV-mRNA test should be used as a complementary tool to a positive HPV DNA test, in order to improve specificity

There are limited available data in the literature regarding HPV-DNA test comparisons between pregnant and non-pregnant populations, providing quite inadequate information in order to draw safe conclusions. Although it is known that pregnancy does not alter the disease progress of HPV infection to cervical cancer [1,5,8-10], most previously reported studies support that HPV-DNA detection rates are higher during pregnancy and HR-HPV genotypes are most common in pregnant women [14,15], as well. There is also controversial information referring to the correlation between gestational age and HPV-DNA detection rates from published literature [13,15]. A plausible explanation suggests that a large number of risk factors are associated with HPV-DNA detection, which must be exactly the same between those two groups in order to examine pregnancy as a unique risk factor for HPV-DNA detection. Thus, further studies examining the comparison between pregnant and non-pregnant populations should be conducted to define and interpret the association of HPV-DNA detection during pregnancy.

Another point of interest that several studies assessed and commented on over the years is HPV-DNA test integration into cervical cancer screening during pregnancy. Despite the fact that the HPV-DNA test is the most reliable and sensitive test in the diagnosis of HPV infection, Khanuja et al. [16], taking into consideration both the high cost of the HPV-DNA test and Pap smear reliability to diagnose HPV in pregnant women at the point of the clinically infective stage, summed up that the Pap smear is more suitable for cervical cancer screening in clinical routine. Moreover, He et al. proposed that pregnant women diagnosed with CIN lesions should be monitored with the HPV-DNA test during pregnancy and postpartum [3]. Nevertheless, a colposcopy-guided cervical biopsy remains the “gold standard” for treatment management. The findings of the study conducted by Lu et al. support that Pap smear results are more representative to decide the clinical management of pregnant women regarding HPV [12] while the value of a positive HPV-DNA test should be restricted to compose an indication for closer follow-up.

Furthermore, Frega et al. and Yang et al. evaluated the accuracy of the HPV-DNA and HPV-mRNA molecular tests in pregnant women with CIN II/CIN III lesions and the postpartum period [17,18]. Both studies found that the HPV-mRNA test has higher specificity than the HPV-DNA test in HPV detection. An explanation was presented for this statement, based on the hypothesis that the HPV-mRNA test has higher specificity due to the fact that it detects a small number of HPV genotypes [17,21]. Finally, Frega et al. claimed that HPV-mRNA might be a useful tool for risk stratification [17], and Yang et al. presented a similar idea supporting that HPV-mRNA could be a complementary tool to a positive HPV-DNA test in pregnant women, in order to improve its specificity and why not to promote triage for colposcopy [18]. Nevertheless, only those two studies evaluated and provided data regarding the sensitivity and specificity of the HPV-DNA test during pregnancy [17,18], a fact that helps the clinical doctor form an initial point of view and highlights the need for studies further investigating this field in order to draw safe conclusions.

## Conclusions

In conclusion, the HPV-DNA test is a useful tool regarding cervical cancer screening in the general population. However, studies that evaluated the HPV-DNA test results in pregnant women compared to non-pregnant showed ambiguous results and further investigation should be conducted to draw safe conclusions. The HPV-DNA test during pregnancy and postpartum might be utilized as a tool for monitoring, risk stratification, and triage of those cases potentially requiring colposcopy, either alone or in combination with the HPV-mRNA test. The Pap smear remains the first line diagnostic tool and colposcopy-guided cervical biopsy remains the “gold standard” for CIN treatment management.
